# Measuring Health Related Quality of Life (Hrqol) in Renal Transplant Patients: Psychometric Properties and Cross-Cultural Adaptation of Kidney Transplant Questionnaire (Ktq-25) in Persian

**DOI:** 10.5812/numonthly.1382

**Published:** 2012-09-24

**Authors:** Ali Tayyebi, Afsaneh Raiesifar, Soheil Najafi Mehri, Abbas Ebadi, Behzad Einolahi, Shadi Pashandi

**Affiliations:** 1 Faculty of Nursing, Baqiyatallah University of Medical Sciences, Tehran, IR Iran; 2 Nephrology and Urology Research Center, Baqiyatallah University of Medical Sciences, Tehran, IR Iran; 3 Bam Nursing Faculty, Kerman University of Medical Sciences, Bam, IR Iran

**Keywords:** Quality of Life, Kidney Transplantation, Questionnaires, Psychometrics

## Abstract

**Background:**

Different measuring tools have been used to understand the outcomes of renal replacement therapies. The goal of renal transplantation is both to ensure survival, and to promote quality of life in the patients. One of the widely used disease-specific instruments to measure the quality of life is the Kidney Transplant Questionnaire (KTQ-25).

**Objectives:**

The current study aimed to perform a cross-cultural adaptation and assess the psychometric properties of the KTQ-25 to Persian.

**Materials and Methods:**

The KTQ-25 was trasnlated according to International Quality of Life Assessment (IQOLA) translation methodology. Cronbach’s alpha coefficient, and test-re-test were used to determine internal consistency, and reliability respectively.

**Results:**

In the test–re-test reliability of all questionnaire items, Pearson correlation was r = 0.96 (P < 0.001). Cronbach’s alpha coefficient estimated the internal consistency for each scale andalpha equal or more than 0.73 was considered satisfactory. Criterion-related validity, measured by the correlation coefficients between the KTQ-25 and the SF-36 Health Survey, was r = 0.63 (P < 0.001).

**Conclusions:**

The psychometric properties of the Kidney Transplant Questionnaire (KTQ-25) in Persian have proven to be satisfactory, therefore the application of this questionnaire in clinical practice can be recommended.

## 1. Background

Patients suffering from end-stage renal disease (ESRD ) need the renal replacement therapies to keep function of kidneys (1, 2). Although, there are medical and surgical treatment modalities, each of them has its advantages, disadvantages, and different levels of impacts on patients’ physical, psychological and social health. Therefore, they may change the health related quality of life (HRQoL) (3).

In the past three decades, advances in immunosuppressive therapy and organ transplant technology have improved patient and graft survival rates in renal transplant recipients (4, 5). The goal of renal transplantation is both to ensure survival, and to promote quality of life in the patients (6). Although, clinicians have relied on the graft survival to determine the efficacy of the transplantation, such measures do not fully address the patient’s general or disease-specific health status. It should be realized that renal transplantation is likely to affect not only patients’ physical well-being but also their social and psychological well-being (7). Renal transplantation patients may have medical complications both in short and long terms. Therefore, in order to prevent aggravations of disease and policy making, health care providers need to assess and interpret the HRQoL continuously (8).

Different measuring tools have been used to understand the outcomes of renal replacement therapies, including mortality, residual renal function, cardiovascular function, malnutrition, psychological status and quality of life. However, survival, cost-effectiveness and quality of life are the main parameters to evaluate treatment of end-stage renal disease (9). HRQoL is a good predictor of mortality for ESRD patients (10). It has been indicated that health related quality of life in patients with successful renal transplantation is more than in patients who use dialysis and are candidates for the renal transplant (11). Along with survival, HRQoL of patients is an important indicator of the effectiveness of the medical care that they receive and is a valid outcome measurement (12).

Generally, quality of life assessment helps to plan the individual treatment strategies, to determine the efficacy of medical interventions, and to evaluate the quality of medical care (13). Over the past 20 years, more instruments have been developed to measure quality of life (14). At the time, there are two main types of HRQoL instruments, including general and disease specific instruments. Disease-specific instruments focus on the domains most relevant to the disease or the condition under study and on the characteristics of patients in whom the condition is most prevalent. Disease-specific instruments are most appropriate for clinical trials in which specific therapeutic interventions are being evaluated (15).

As defined by the World Health Organization, quality of life is an individual’s perception of his position in life in the context of the culture and value systems in which he lives with the relation to his goals, expectations, standards, and concerns (16). One of the widely used disease-specific instruments to measure the quality of life is the Kidney Transplant Questionnaire (KTQ-25) (17).

## 2. Objectives

Although the number of patients on the waiting lists is progressively growing in the world. In Iran the donor rate has increased over the past 10 years, from less than 1% by the end of year 2000 to almost more than 16.3% of kidney transplantations to date (18), but there is not any specific and valid Persian questionnaire to follow up the quality of life. Therefore, the current study meant to perform a cross-cultural adaptation and to assess the psychometric properties (reliability and validity) of the KTQ-25 translation to Persian and also, to measure the health related quality of life in Iranian renal transplantation patients.

## 3. Materials and Methods

### 3.1. Questionnaire

The KTQ-25 was developed by Laupacis et al. (1993) with the involvement of renal transplant patients and clinical experts. 25 items are classified in 5 domains: Physical symptoms, Fatigue, Uncertainty/fear, Appearance and Emotions. Responses are obtained on a 7 point Likert scale. The lowest score represents the lowest quality of life. For the analysis, all scores in each domain were added and divided by number of items in that dimension (19).

### 3.2. Translation

Permission for translation was asked from Laupacis et al. Translation was made according to International Quality of Life Assessment (IQOLA) translation methodology (20).

For this purpose, two experts translated the original English version to Persian independently, then an expert group including 30 persons (nephrologists, psychologist, registered master and PhD nurses) revised the two documents primarily and final provisional translation was provided (forward step). After that, the prepared questionnaire was translated to the original language (English) by two experts whose native languagewas English (backward step). After the revision, the two prepared questionnaires were checked for differences by the expert group. By a careful review and cultural adaptation, few changes were made specially in the questionnaire format. Subsequently the provisional forward translation questionnaire was piloted by 25 kidney transplant patients who had signed for informed consent and completed the questionnaire.

### 3.3. Data Collection and Statistical Analysis

By methodological research design the KTQ-25 was applied to 220 subjects of kidney transplant patients aged 15 and more. According to the IQOLA Project to test psychometric properties of the KTQ-25 Iranian version, several tests were performed. Cronbach’s alpha coefficient, and test-re-test were used to determine internal consistency, and reliability respectively. Validity was assessed by known groups comparison test. Also, criterion-related validity was performed by comprised to Health Survey Questionnaire Short form (SF-36) Iranian version results.

## 4. Results

Patient characteristics are presented in Table 1. Two of 220 patients excluded because of defect on completing the questionnaire therefore final analysis was performed on 218 subjects. The mean age of the sample group was 41.24 ± 13.93 years old. The subjects consisted of 58.7% male who were mostly married.

**Table 1:** Sociodemographic and Clinical Characteristics of the Sample

Demographic, Variables	No. (%)
**Age, y**	
≤ 24	31 (14)
25-44	98 (46)
45-64	77 (35)
≥65	11 (5)
Total	217 (100)
**Job Status**	
Unemployment	61 (29)
Free job	49 (32)
Clerk	28 (13)
Housewife	54 (26)
Other	19 (10)
Total	211
**Education Grade**	
Primary	73 (35)
Secondary	51 (24)
Diploma	58 (27)
Academic	30 (14)
** Clinical Variables**	**No. (%)**
**Dialysis, y**	
No dialysis	55 (25)
< 1	100 (46)
1-2 y	34 (16)
> 2	28 (13)
**ESRD Causes**	
Hypertension	60 (30)
Unknown	46 (22)
Congenital disease	28 (13)
Diabetes mellitus	23 (11)
Other causes	50 (24)
**Donner types**	
Relative	11 (5)
Un-relative	183 (84)
Cadaver	23 (11)
**Transplantation, y**	
< 1	46 (22)
1-5	79 (39)
6-10	60 (30)
11-15	14 (6)
> 15	7 (3)

Abbreviation: ESRD, end stage renal diseases

Although, there were no difficulties in the items translation to Persian, there was the same definition for two words including “difficulty and distress” in Persian; therefore, all likert scales were modified to severity determination. In order to make it to complete the Persian version of questionnaire, the general format was changed to self-reported format. The mean time to complete the KTQ-25 was about 13 minutes and more than 90% of subjects stated that they did not have any difficulties in completing the questionnaire. To test the reliability of the internal consistency for each scale Cronbach’s alpha coefficient estimation was used and alpha equal to or greater than 0.73 was considered satisfactory. In test–re-test reliability of all items of questionnaire, pearson correlation was r = 0.96 (P < 0.001).

The descriptive statistics for the five dimensions of the KTQ-25 and eight dimensions of SF-36 are presented in Table 2. The correlation coefficients among the dimensions of the KTQ-25 and intra scale correlation, which evaluates the construct validity, are presented in Table 3. The coefficients ranged between the minimum of 0.73 obtained for “Appearance”, and the maximum of 0.86 obtained for “Physical symptoms”. Criterion-related validity, measured by the correlation coefficients between the KTQ and the SF-36 Health Survey, was r = 0.63 (P < 0.001). Correlation coefficients between the KTQ and two domains of the SF-36 dimensions are presented in Table 4.

**Table 2:** The Scores of the HRQoL Questionnaires: Kidney Transplant Questionnaire and SF-36 Health Survey

Questionnaire Dimentions	Scores, Mean ±SD
**KTQ-25**	
**Physical symptoms**	4.5 ± 1.5
**Fatigue**	4.7 ± 1.6
**Uncertainty/Fear**	4.5 ± 1.8
**Appearance**	5.7 ± 1.4
**Emotion**	4.8 ± 1.6
**Total Score**	4.9 ± 1.2
**SF-36**	
**Physical function**	65.5 ± 25.6
**Role physical**	46.6 ± 36.9
**Bodily pain**	66.2 ± 28.8
**General health**	61.0 ± 21.2
**Vitality**	59.7 ± 23.2
**Social function**	64.6 ± 25.0
**Role emotional**	53.9 ± 37.1
**Mental health**	63.2 ± 22.6
**Total Score**	60.6 ± 18.7

**Table 3:** Reliability: Cronbach’s Alpha Coefficient and Intra Class Correlation Coefficient^a^

KTQ Dimensions	Cronbach’s α	Intra Scale Correlation
**Physical symptom**	0.86	0.69
**Fatigue**	0.85	0.88
**Uncertainty/Fear**	0.79	0.82
**Appearance**	0.73	0.63
**Emotion**	0.84	0.86
**Total**	0.93	

^a^P < 0.001

**Table 4:** Correlation Coefficients (r) Between the KTQ Dimensions and Two Dimensions of SF-36^a^

**KTQ Dimensions SF-36 Dimensions**	**Physical Symptoms**	**Fatigue**	**Uncertainty/ Fear**	**Appearance**	**Emotion**
**Physical health**	0.47	0.59	0.47	0.18	0.52
**Mental health**	0.48	0.63	0.53	0.19	0.62

^a^P < 0.001

Known groups comparison to test the scale validity, was also performed. It hypothesized that women would have poorer health status than men. The analysis showed that the females significantly had lower scores in all measures as expected (P < 0.001). This indicated that the KTQ-25 well discriminated between sub-groups of people who differed in gender.

Performing the factor analysis, KMO = 0.88, and Bartlett’s test (P < 0.001) indicated that the sample size was adequate. The principal component analysis with rotation solution (Varimax method) performed, 7- factor was extracted, and cumulative variance was 63.41.

## 5. Discussion

The psychometric properties of the Kidney Transplant Questionnaire (KTQ-25) in Persian have proven to be satisfactory, therefore the recommendation of the use of this questionnaire in clinical practice is allowed.

This is the first specific instrument which is culturally adjusted and validated in Persian. Because of the shortage of available patients with a certain disease, the sample size of studies which evaluate the psychometric properties of specific HRQoL assessment instruments, could never reach the magnitude of the validations of generic instruments (15), but, in this study, a large sample size was used, whereas the study of validation of the original version of the Kidney Transplant Questionnaire was carried out with a sample size of only 26 kidney transplant patients and Spanish validation was also carried out with only 45 patients (15). Therefore, the results of this study can be considered as Iranian kidney transplant normative data for the KTQ-25 and might be used in the future studies of renal transplant patients.

Generally, all psychometric tests of the KTQ-25 Iranian version indicated satisfactory results. The feasibility of the questionnaire was good, as indicated by the low number of items not answered or not understood by the interviewees, also by only 13 minutes average time of administration. The short time required makes the questionnaire suitable for everyday clinical use, as well as this study, which in most cases the questionnaire was self- completed after briefing instruction.

Reliability of the questionnaire as measured by the Cronbach’s alpha for all five scales was very good and similar to the Spanish version. Intra scale correlation of the KTQ was good, conferring the instrument adequate construct validity. These results were similar to original and Spanish version (15, 21). The positive correlation coefficients found between the dimensions of the KTQ and the SF-36, demonstrated that both instruments evaluate the same concept.

The feasibility, validity, reliability and sensibility to change, of the Iranian version of the Kidney Transplant Questionnaire-25 are similar to those of the original instrument. Thus, a specific HRQoL assessment instrument is available now in the Persian language. This instrument will be useful for the individual evaluation of patients with end-stage renal disease who receive a kidney transplant, also to evaluate the different types of immunosuppressive , and other types of therapies, which influence the evolution of the kidney transplant.
